# Perception and Awareness about Developmental Dysplasia of the Hip in Children among Pregnant Ladies in the Aseer Region, Southwestern Saudi Arabia

**DOI:** 10.3390/healthcare9101384

**Published:** 2021-10-16

**Authors:** Mahdi M. Alqarni, Ayed A. Shati, Youssef A. Al-Qahtani, Wafaa S. Alhifzi, Wael S. Alhifzi, Rasha S. Al Saleh, Nada A. Alqahtani, Mohammed A. Alshehri

**Affiliations:** 1 Department of Pediatric Orthopedics, Abha Maternity and Children Hospital, Abha 62529, Saudi Arabia; dralqarnim@gmail.com; 2 Department of Child Health, College of Medicine, King Khalid University, Abha 62529, Saudi Arabia; youssef9811@hotmail.com; 3 Medical Interns, College of Medicine, King Khalid University, Abha 62529, Saudi Arabia; walhifzi@hotmail.com (W.S.A.); Rasha_44@hotmail.com (R.S.A.S.); nada.hanamichi@gmail.com (N.A.A.); mohammad666zx@gmail.com (M.A.A.); 4 Orthopaedic Resident, Department of Orthopedic Surgery, Aseer Central Hospital, Abha 62523, Saudi Arabia; alhifzi@mail.com

**Keywords:** developmental dysplasia, hip joint, congenital anomalies, children, pregnant ladies, perception

## Abstract

**Background:** Developmental dysplasia of the hip (DDH) is classified as a group of malformations, varying from abnormal acetabulum (dysplasia) and mild subluxation of the femoral head to fixed displacement (congenital dislocation). This study aimed to assess the knowledge level and its determinants regarding DDH in children among pregnant females in the Aseer region of southwestern Saudi Arabia. **Methods:** A descriptive cross-sectional study was conducted targeting all pregnant females in the Aseer region between 1 February 2021 and 1 May 2021. A pre-structured online questionnaire was constructed by the researchers to obtain the participating females’ bio-demographic data (including age, education status, and obstetric history) and awareness regarding DDH. The last section asked for their source of information regarding DDH. **Results:** A total of 253 pregnant females (aged between 18 and 45 years; mean age = 30.5 ± 10.2 years) fulfilling the inclusion criteria completed the study questionnaire. About 5% of the females reported having a child with DDH, and 166 (65.6%) pregnant females knew about DDH. Additionally, 110 (43.5%) females reported that they know about how DDH is treated, and 99 (39.1%) knew about DDH complications. The most commonly reported source of information was relatives and friends (44.3%), followed by social media (11.9%) and study and work (7.1%). **Conclusions:** Pregnant females in the Aseer region have poor knowledge and awareness about DDH and its causes, treatment modalities, and complications. Higher knowledge was associated with either high parity or having a child with DDH.

## 1. Introduction

Developmental dysplasia of the hip (DDH) is defined as abnormal formation of the hip joint where the femoral head is not stable within the acetabular socket, which is its normal articulating site. This abnormal formation leads to dislocation/subluxation of the hip joint [1]. The exact etiology of DDH remains debatable since it is believed to be a ‘multifactorial trait’ where many factors are implicated [2].

The incidence of DDH varies depending on several factors. An estimated 1 in 1000 children is born with a dislocated hip, and 10 in 1000 may have hip subluxation [3,4,5]. The most prominent risk factors for DDH include abnormal intrauterine position (breech presentation), female sex, race, positive family history, firstborn status, and oligohydramnios [6,7,8,9]. 

The clinical presentation of DDH ranges from subtle hip dysplasia to reducible subluxation/dislocation and, eventually, irreducible hip joint dislocation [10]. Furthermore, neurological, neuromuscular, idiopathic, and skeletal dysplasias are excluded from DDH, as hip malformation is due to congenital malformation of the developing hip joint. Conventionally, reducibility of the dislocated hip is the most significant outcome measure. Clinically, DDH can be diagnosed when the leg on the affected side appears shorter and is turned outward of the dislocated hip and the skin of the thigh appears uneven; additionally, the hip looks wider than normal. The diagnosis can be confirmed radiologically using X-ray or ultrasound [11,12,13].

The treatment of DDH depends on the clinical presentation, age of the child, and their general health. The treatment focuses mainly on reducing the femoral head into its normal site within the acetabulum. Multiple treatment modalities are available; these include using a brace or harness, which is most often used, whereas casting and surgery are the last choice if all treatment fails [14].

The incidence of DDH in the Aseer region of Saudi Arabia is around 3.5 per 1000 live-births; consequently, it is a major health concern for pediatric orthopedic surgeons [15]. Around 30% of DDH cases in Saudi Arabia require surgical intervention due to late reporting and poor awareness [16]. It has also been highlighted that knowledge and awareness about DDH, even among healthcare providers, are not adequate and require substantial efforts for upgradation [17]. This may further result in a failure to educate mothers regarding DDH. Over time, the significance of improving awareness among expecting mothers about DDH is rooted in the fact that a higher incidence of DDH is associated with degenerative hip disorders, which are a primary cause of early hip arthritis [18].

Therefore, this study aimed to assess the knowledge and perception level regarding DDH among pregnant females in the Aseer region. We also aimed to assess the determinants that affect their level of awareness as well as the source of their information. 

## 2. Methods

A descriptive cross-sectional study was conducted targeting all pregnant females who visited the Abha Maternity and Children Hospital in the Aseer region of southwestern Saudi Arabia for routine antenatal care between 1 February 2021 and 1 May 2021. Ethical approval for the study was obtained from the Regional Committee for Research Ethics (H-06-B-091) in the Aseer region with the record number 2-3-2021. Written informed consent was obtained from all pregnant females who agreed to participate. 

A pre-structured online questionnaire was developed in the native language by the authors after intensive review of the literature and expert consultation for relevant items. After constructing the questionnaire, three subject experts reviewed the items independently to assess their content validity and applicability. All suggested modifications were applied until reaching the final form. Subsequently, a pilot study was conducted on 15 females (who were then excluded from the final study) to assess the tool’s clarity and reliability. The participants were able to complete the questionnaire within 20 min; a reliability coefficient (Cronbach’s α) of 0.74 was obtained for the questionnaire. 

The questionnaire consisted of two main sections. The first section collected information regarding demographic and obstetric data, such as age, educational status, and obstetric history, such as parity. The second part of the questionnaire included nine questions that were directed to assess the participant’s awareness and knowledge about the complications and risk factors associated with DDH. All the questions were close-ended.

### Data Analysis

After extracting all the relevant data, they were revised, coded, and fed into the Statistical Package for Social Sciences (SPSS) software, version 22 (IBM Corp, Chicago, IL, USA). 

For the awareness items, each correct answer was scored one point, and the total summation of the discrete scores of the different items was calculated. A patient with a score less than 60% (6 points) of the maximum score was considered to have poor awareness, while a score of 60% (7 points or more) of the maximum or more indicated good awareness. Descriptive analysis based on frequency and percentage was performed for all variables, including the females’ demographic data, gravidity, parity, awareness regarding DDH, and having a child with DDH. Cross-tabulation was used to assess the distribution of awareness level regarding DDH based on their personal obstetric data and source of information. To measure the significance association, we used the chi-square test at a 5% level of significance (*p* < 0.05).

## 3. Results

A total of 253 pregnant females aged between 18 and 45 years, with a mean age of 30.5 ± 10.2 years, who fulfilled the inclusion criteria completed the study questionnaire (Table 1). Of these, 182 (71.9%) females were university graduates. Regarding gravidity, 60 (23.7%) females were in their first pregnancy, while 140 (55.3%) were in their fourth pregnancy or more. When considering parity, 98 (50.8%) females were nulliparous, while 54 (28%) were primipara. Only 14 (5.5%) females reported having a child with DDH, of which surgical intervention was the modality of treatment in eight (57.1%) patients, and four (28.6%) received conservative treatment, while two (14.3%) did not receive any treatment. 

**Figure 1:**
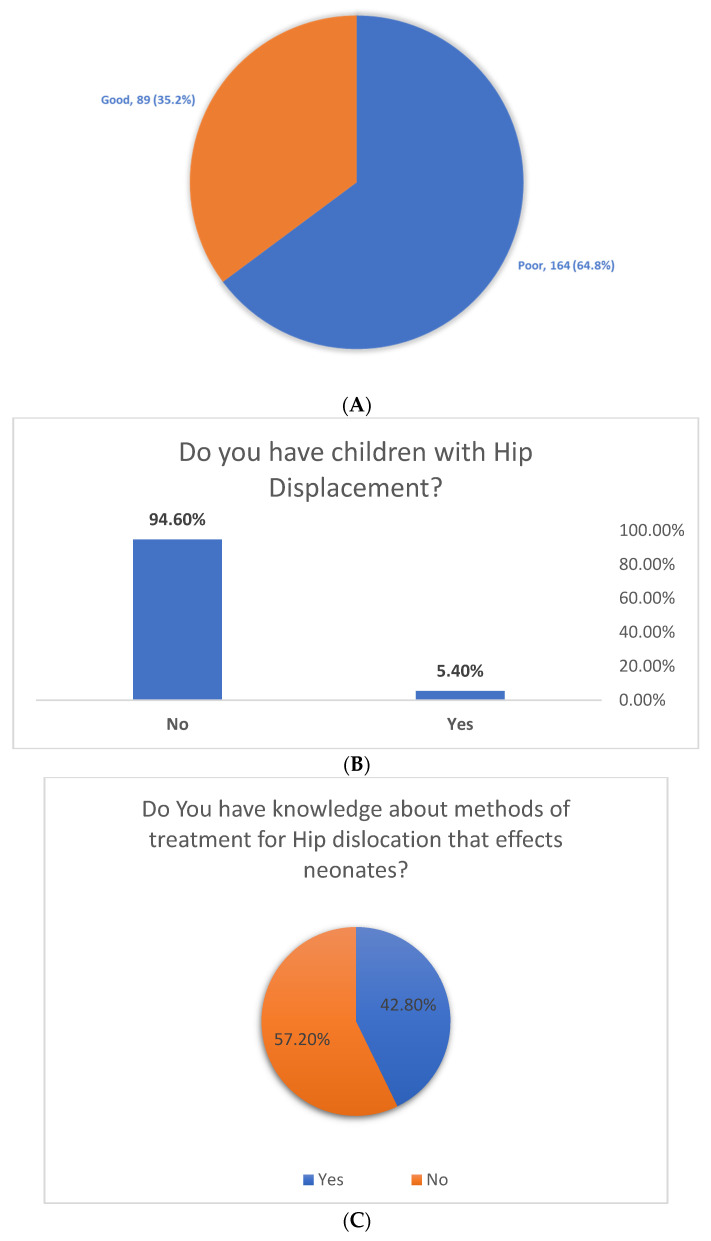
(**A**) Overall awareness level regarding DDH among pregnant females in the Aseer region, Saudi Arabia. (**B**) Do you have children with hip displacement? (**C**) Do you have knowledge about methods of treatment for hip dislocation that affects neonates?

In total, only 89 (35.2%) females had good awareness levels regarding DDH (Figure 1A). According to Figure 1B, 5.4% of the respondents have children with hip displacement (Figure 1B). Figure 1C depicts that 42.8% said ‘yes’ when asked if they had knowledge about methods of treatment, while 57.2% had no idea about methods of treatment (Figure 1C).

Table 2 presents the awareness results regarding DDH in the Aseer region of Saudi Arabia. It was found that 166 (65.6%) pregnant females knew about DDH. Furthermore, 110 (43.5%) females stated that they knew about the treatment of DDH, while only 99 (39.1%) females were aware of the complications of DDH. 

As for the causes of DDH, 141 (55.7%) females reported knowing the causes; among those, 108 (76.6%) selected the mode of delivery option, 21 (14.9%) selected genetic causes with family history, and 9 (6.4%) selected the ‘breech presentation during pregnancy’ option. A total of 220 females were incorrect in agreeing that the mode of delivery may be associated with DDH. As for the name of the condition, 13 (5.1%) knew the correct name (developmental dysplasia of hip joint). Additionally, 192 (75.9%) females reported that they could identify a child with a dislocated hip due to his gait, while 200 (79.15%) females incorrectly reported that hip dislocation is painful for the child. 

Table 3 lists the various sources of information about DDH as reported by the participating pregnant females. The most commonly reported source of information was relatives and friends (50.0%), followed by social media (36.7%) and studies and work (94.4%), while 3.4% of the participating females had no specific source for their information. 

Table 3 also reveals the pattern of distribution of awareness level among pregnant females regarding DDH according to their bio-demographic data. Almost 50% of multiparous females (3+ parity) had a good awareness level regarding DDH compared to the 38.8% nulliparous females; this difference was found to be statistically significant (*p* = 0.049). Additionally, 71.4% of the females having a child with DDH had good awareness levels regarding the condition in comparison to the other 33.1% (*p* = 0.003). Overall, 94.4% of females who obtained information from their studies or job had good awareness levels compared to the 3.4% of participants with no reported source (*p* = 0.001). Other factors, including age and gravidity, had no significant relation with females’ awareness level.

## 4. Discussion

The current study aimed to assess the knowledge and perception among pregnant women of the Aseer region regarding DDH. Additionally, the authors aimed to investigate the relationship between education levels and knowledge of DDH, as well as the association between the number of pregnancies and knowledge of DDH. DDH is a common, yet preventable, reason for childhood disability. Unfortunately, a delayed diagnosis of the condition means that the child is more likely to require surgery, which is associated with a higher risk of long-standing complications. Despite the recent introduction of national-level screening programs, which in some cases also include universal screening of newborns, late diagnosis of the condition still hampers the aim to reduce occurrence [19,20]. 

Our results revealed that about one-third of the participating females had good awareness regarding DDH. Although two-thirds of the participating females reported that they knew about DDH, less than 50% were aware of its treatment modalities, and about one-third knew about the complications of DDH. More than half of the respondents knew the reasons for DDH, but ~75% of these females incorrectly mentioned mode of delivery as the main cause, while only 15% of females reported family history, which is one of the leading risk factors for DDH.

Despite a lack of knowledge, three-fourths of the participating females could identify a child with a dislocated hip from his gait, while the majority of females (>75%) incorrectly believed that hip dislocation is painful for the child. These discrepancies in the knowledge level can be explained partially by the fact that one-third of the participating females had no source of information, while relatives were the main source among 44% of the females, followed by social media; notably, study or work experience in the healthcare field had very little contribution as a source of information. A higher level of knowledge was detected among multiparous females who had a child with DDH and those who obtained their information from study or work in the medical field. Almahdi et al. [20] also reported that the awareness level among females regarding DDH was poor. Furthermore, this lack of awareness regarding the treatment modalities often leads to late diagnosis, which can become a major health concern as it increases the chances of surgery and associated morbidity. 

Conventional practices regarding neonatal care may also play an important role in the progression of DDH. One such practice, which is commonly carried out in the Middle East, is swaddling [21]. A majority of the sampled Saudi females learned how to swaddle from their family or friends, while only 4% learned from healthcare providers. More than 77% of the participants were not aware of the negative effects of swaddling on children’s hips, and around 63% did not know the correct swaddling method. About 7% of them said they would use swaddling despite knowing the possible harm to the child. Recent studies have pointed out that traditional swaddling techniques with restricted leg movements can potentially harm unstable hips, especially in infants who are undergoing treatment for hip dysplasia. It is, therefore, important to educate parents and families about the correct swaddling technique, more so if the infant is prone to or suffering from dysplasia of the hips [22].

Some studies assessed the awareness regarding DDH in community categories other than females. Uzel et al. [23,24] found that only 27.5% of physicians were aware of incorrect traditional attitudes and practices which were potential risk factors for DDH. However, a later test following an awareness program revealed that the awareness levels increased to 81.4%. This improvement is indicative of the importance of awareness programs. Healthcare providers play an important role in educating members of society; therefore, enhancing and upgrading their knowledge and awareness will be directly beneficial to improve the awareness among expecting mothers. Melo TE et al. [25] also assessed the professionals involved in diagnosing DDH on their knowledge about the condition and found that 50% of these professionals had not examined any DDH case in the last year. As for self-assessed knowledge measured on a scale of 0–10, the average score was 4.25. The most recognized and neglected risk factors were pelvic presentation (68%) and congenital muscular torticollis (CMT) (9.3%), respectively. None of the participants could identify all the risk factors, and the average number of risk factors identified was two. Nearly 74% of the participants did not know that the time of birth is the ideal moment for diagnosis; only 17% reported the ideal time as after the first month. Regarding neglected severe DDH, 45.3% of the participants failed to recognize its natural history [25]. 

Our study had some limitations as it was limited to only one center and one geographical location. However, our results highlight the importance of healthcare providers educating mothers regarding the necessary steps to prevent complications. Moreover, this could encourage other researchers to conduct similar studies. 

## 5. Conclusions

The current study revealed that although the pregnant females in the Aseer region were aware of DDH, their knowledge regarding its treatment, causes, complications, and risk factors was poor. Greater awareness was associated with either high parity or having a child with DDH. Additionally, study or work experience in the medical field had a significant role in enhancing their awareness of the disease, irrespective of having a lower rank among the sources of information. It is important to educate all community members, especially pregnant women, regarding DDH as it is a preventable disease but may have lifelong implications. Special attention should be paid to educating mothers of high-risk babies to promote early detection and management. 

## Figures and Tables

**Table 1:** Bio-demographic data of participating pregnant females.

Bio-Demographic Data	Frequency	Percentage (%)
**Age (in years)**		
<25	58	22.9%
25–30	52	20.6%
31–35	36	14.2%
36–40	39	15.4%
41–45	68	26.9%
**Educational level**		
Secondary/below	71	28.1%
University/above	182	71.9%
**Gravidity**		
Primigravida	60	23.7%
2nd pregnancy	18	7.1%
3rd pregnancy	35	13.8%
4th/more	140	55.3%
**Parity**		
Nullipara	98	50.8%
1 time	54	28.0%
2 times	23	11.9%
3/more times	18	9.3%
**If yes, type of received care**		
Conservative treatment	4	28.6%
Surgical intervention	8	57.1%
Nothing	2	14.3%

**Table 2:** Awareness levels regarding developmental dysplasia of the hip joint in pregnant females of the Aseer region, Saudi Arabia.

Awareness Items	No	%
**Know about developmental dysplasia of the hip**		
Yes	166	65.6%
No	87	34.4%
**Know how DDH is treated**		
Yes	110	43.5%
No	143	56.5%
**Know about DDH complications**		
Yes	99	39.1%
No	154	60.9%
**Know causes of DDH**		
Yes	141	55.7%
No	112	44.3%
**If yes, mention (n = 141)**		
Genetic causes with family history	21	14.9%
Mode of delivery	108	76.6%
Child gender	3	2.1%
Setting position during pregnancy	9	6.4%
**Mode of delivery related to DDH**		
Yes	220	87.0%
No	33	13.0%
**Can you identify a child with a dislocated hip from their gait?**		
Yes	192	75.9%
No	61	24.1%
**Hip dislocation causes pain to the child**		
Yes	200	79.1%
No	53	20.9%

**Table 3:** Distribution of awareness level among pregnant females regarding DDH by their bio-demographic data.

Factors	Awareness Level				*p*-Value
	Poor		Good		
	No	%	No	%	
**Age (in years)**					0.498
<25	36	62.1%	22	37.9%	
25–30	38	73.1%	14	26.9%	
31–35	25	69.4%	11	30.6%	
36–40	22	56.4%	17	43.6%	
41–45	43	63.2%	25	36.8%	
**Educational level**					0.145
Secondary/below	51	71.8%	20	28.2%	
University/above	113	62.1%	69	37.9%	
**Gravidity**					0.607
Primigravida	38	63.3%	22	36.7%	
2nd pregnancy	14	77.8%	4	22.2%	
3rd pregnancy	24	68.6%	11	31.4%	
4th/more	88	62.9%	52	37.1%	
**Parity**					0.049 *
None	60	61.2%	38	38.8%	
1 time	43	79.6%	11	20.4%	
2 times	14	60.9%	9	39.1%	
3/more times	9	50.0%	9	50.0%	
**Have child with DDH**					0.003 *
Yes	4	28.6%	10	71.4%	
No	160	66.9%	79	33.1%	
**Source of information**					0.001 *
None	84	96.6%	3	3.4%	
Relatives	56	50.0%	56	50.0%	
Social media	19	63.3%	11	36.7%	
Study/work	1	5.6%	17	94.4%	
Others	4	66.7%	2	33.3%	

*p*-value obtained from the Pearson X^2^ test; * *p* < 0.05 (significant).

## Data Availability

The data used and/or analyzed during the current study are not publicly available. They can be obtained from the corresponding author on reasonable request.

## List of Abbreviations

DDH	Developmental dysplasia of the hip
SPSS	Statistical Package for Social Sciences
